# Vertical transmission of the gut microbiota influences glucose metabolism in offspring of mice with hyperglycaemia in pregnancy

**DOI:** 10.1186/s40168-022-01318-8

**Published:** 2022-08-09

**Authors:** Cunxi Xue, Qinyuan Xie, Chenhong Zhang, Yimeng Hu, Xiaoting Song, Yifan Jia, Xiaoyang Shi, Yiqi Chen, Yalei Liu, Lingyun Zhao, Fenglian Huang, Huijuan Yuan

**Affiliations:** 1 Department of Endocrinology of Henan Provincial People’s Hospital, Henan Provincial Key Laboratory of Intestinal Microecology and Diabetes Control, People’s Hospital of Zhengzhou University, Zhengzhou, Henan China; 2 State Key Laboratory of Microbial Metabolism, School of Life Sciences and Biotechnology, Shanghai Jiao Tong University, Shanghai, China

**Keywords:** Vertical transmission, Hyperglycaemia in pregnancy, Glucose metabolism, Microbiome, Metabolites, Multiomics analysis

## Abstract

**Background:**

Hyperglycaemia in pregnancy (HIP) is a common metabolic disorder that not only poses risks to maternal health but also associates with an increased risk of diabetes among offspring. Vertical transmission of microbiota may influence the offspring microbiome and subsequent glucose metabolism. However, the mechanism by which maternal gut microbiota may influence glucose metabolism of the offspring remains unclear and whether intervening microbiota vertical transmission could be used as a strategy to prevent diabetes in the offspring of mothers with HIP has not been investigated. So we blocked vertical transmission to investigate its effect on glucose metabolism in the offspring.

**Results:**

We established a murine HIP model with a high-fat diet (HFD) and investigated the importance of vertical transmission of gut microbiota on the glucose metabolism of offspring via birth and nursing by blocking these events through caesarean section (C-section) and cross-fostering. After weaning, all offspring were fed a normal diet. Based on multi-omics analysis, biochemical and transcriptional assays, we found that the glucometabolic deficits in the mothers were subsequently ‘transmitted’ to the offspring. Meanwhile, the partial change in mothers’ gut microbial community induced by HIP could be transmitted to offspring, supported by the closed clustering of the microbial structure and composition between the offspring and their mothers. Further study showed that the microbiota vertical transmission was blocked by C-section and cross-fostering, which resulted in improved insulin sensitivity and islet function of the offspring of the mothers with HIP. These effects were correlated with changes in the relative abundances of specific bacteria and their metabolites, such as increased relative abundances of Bifidobacterium and short-chain fatty acids. In particular, gut microbial communities of offspring were closely related to those of their foster mothers but not their biological mothers, and the effect of cross-fostering on the offspring’s gut microbiota was more profound than that of C-section.

**Conclusion:**

Our study demonstrates that the gut microbiota transmitted via birth and nursing are important contributors to the glucose metabolism phenotype in offspring.

Video Abstract

**Supplementary Information:**

The online version contains supplementary material available at 10.1186/s40168-022-01318-8.

## Introduction

Among adolescents and young adults, the prevalence of type 2 diabetes mellitus (T2DM) is increasing precipitously, and HIP is one of the most important risk factors for T2DM development among the respective offspring [1]. HIP is a very common metabolic disorder during pregnancy that consists of two categories: pregestational diabetes (PGDM) and gestational diabetes mellitus (GDM) [2]. It is estimated that in 2021, there were 21 million (16.7%) live births to women who had some form of HIP [3]. HIP not only causes β cell dysfunction and insulin resistance in children [4, 5], but also confers an almost doubled risk of developing T2DM later in life [6, 7]. Identifying the specific mechanism by which maternal HIP affects glucose metabolism in offspring will help develop intervention strategies to prevent T2DM development in offspring.

Emerging evidence indicates that the gut microbiota might be associated with HIP and its adverse effects on offspring. Specifically, women with HIP have gut microbial dysbiosis with lower microbial richness and diversity than healthy women [8]. Importantly, newborns of mothers with HIP showed a significant decrease in alpha diversity [9, 10], increased relative abundance of the phyla Proteobacteria and Actinobacteria and decreased relative abundance of the phylum Bacteroidetes, and they were more predisposed to T2DM development at later stages [9]. In addition, gut microbiota-derived metabolites contribute to offspring outcomes; for example, short-chain fatty acids (SCFAs) supplementation could reduce the susceptibility of offspring to T2DM developed in response to an HFD later in life in the mice [11]. These observations suggest that the maternal gut microbiota shapes the offspring microbiota, which is associated with glucose metabolism of the offspring.

Maternal gut microbiota affects the offspring’s gut microbiota through vertical transmission, and vertical mother-to-infant transmission of the microbiota is influenced by delivery mode and breastfeeding [12–15]. Caesarean section (C-section) blocks the vertical transmission from the mother to the newborn through the birth canal during natural labour [16]. Meanwhile, microbe is one of the natural compositions of breast milk that flows from mother to infant through breastfeeding and the composition of breast milk is also associated with early metabolic functional pathways of the gut microbiota [17, 18]. However, how the mode of delivery and lactation determine the microbiota of offspring and the causality of glucose metabolism is unclear.

In this study, to investigate the effect of vertical microbiota transmission on glucose metabolism of offspring, we built HIP model and applied C-section and/or cross-fostering to block the vertical transmission. We observed that blocking of vertical mother-to-offspring microbial transmission led to an improvement in insulin sensitivity and islet function of the offspring. By assessing changes in the offspring microbiota and faecal metabolites, we showed that microbiota acquired via birth and nursing respectively was significantly different, suggesting that birth and nursing contribute to formate the structure and composition of the gut microbiota and faecal metabolites in offspring. Our findings indicate that a healthy gut microbiome is essential for mothers in pregnancy to avoid disrupted microbiota vertical transmission to their offspring, because the disrupted microbiota may result in impaired glucose metabolism on their offspring. For mothers with HIP, early intervention on offspring’s microbiota (such as supplementation of probiotics or prebiotics) is key to correct abnormal gut microbiota and reduce the risk of T2DM in offspring.

## Results

### Mice with hyperglycaemia in pregnancy affect microbiota alterations and metabolic deficits in offspring

**Fig. 1:**
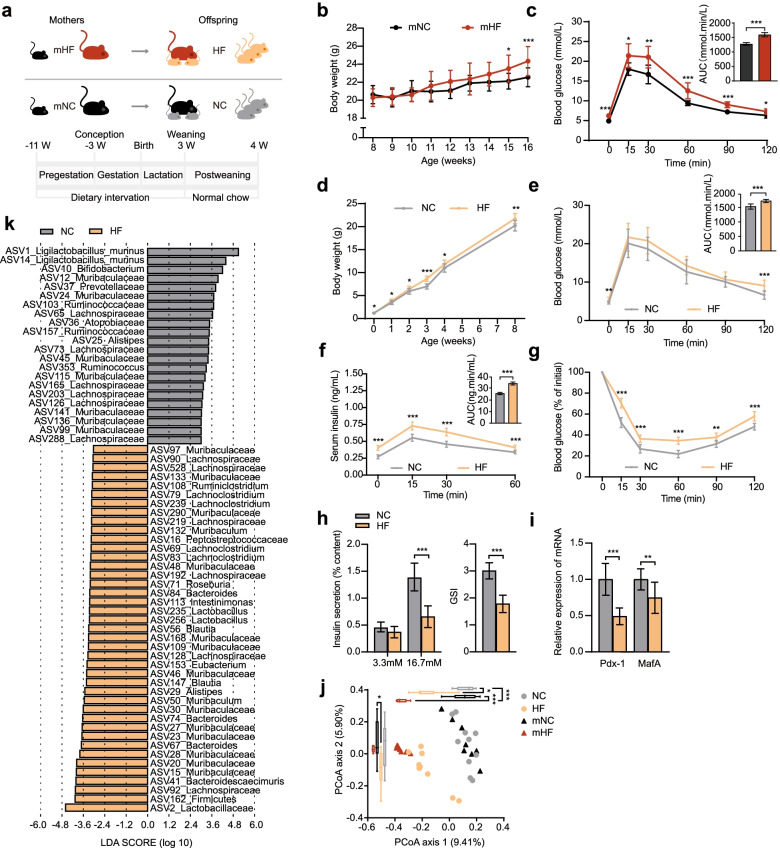
HIP leads to gut microbiota alterations and metabolic deficits in mouse offspring. **a** Hyperglycaemia in pregnancy (HIP) model and experimental scheme of the mice. **b** Body weight and **c** blood glucose levels and areas under the curve (AUCs) during the oral glucose tolerance test (OGTT) of the mHF group and the mNC group. **d** Body weight, **e** blood glucose levels and AUCs during the OGTT, **f** serum insulin levels by ELISA and AUCs during the OGTT and **g** changes in blood glucose levels (% of initial) and AUCs during the insulin tolerance test (ITT) of offspring of the mHF and mNC groups (HF and NC groups, respectively). **h** The amount of insulin secretion upon stimulation with 3.3 mmol/L (low) and 16.7 mmol/L (high) glucose and the glucose stimulation index (GSI) of islets isolated from pancreatic tissues of the offspring of the mHF and mNC groups. **i** Pdx-1 and MafA mRNA expression levels by qPCR in pancreatic tissues of offspring of the mHF and mNC groups. Data are presented as the mean ± SD; *P<0.05, **P<0.01, ***P<0.001. **j** Principal coordinate analysis (PCoA) of the maternal mice and their offspring on the first two principal coordinates was performed based on the Jaccard distance. Comparisons were performed using the Kruskal–Wallis test for significant differences, *P<0.05, **P<0.01, ***P<0.001. **k** Identification of major amplicon sequence variants (ASVs) contributing to the differences in the gut microbiota of the offspring groups using Linear discriminant analysis Effect Size (LEfSe). mHF (*n*=7): maternal group with high-fat diet; mNC (*n*=8): maternal group with normal control diet; HF (*n*=10): offspring delivered by mHF group; NC (*n*=10): offspring delivered by the mNC group

We first developed a HIP mouse model with a high-fat diet (HFD) or a normal chow diet (NCD) for 8 weeks before mating (Fig. 1a). As expected, mice with HFD feeding (the mHF group) had significantly greater weight gain than maternal mice with normal chow diet (NCD) feeding (the mNC group) after 8 weeks HFD (Fig. 1b), coinciding with an increased blood glucose level measured by an oral glucose tolerance test (OGTT) and area under the curve (AUC) value (Fig. 1c), indicating that the HIP model was established successfully. mHF and mNC were then mated with male mice of the same age with NCD feeding, and female mice were maintained on the same diet that they received during their pre-gestational period. All offspring were delivered vaginally and raised by their respective mothers. At weaning (postnatal day 21), all offspring were strained an NCD after weaning (Fig. 1a). Although the body weight of offspring from mHF mice (HF group) was lower than that of offspring from the mNC mice (NC group), it increased rapidly from 1 week of age (Fig. 1d). At 4 weeks of age, the HF group showed a higher glucose AUC value following an OGTT owing to the impairment in glucose clearance ability at 0 and 120 min after glucose injection (Fig. 1e). Compared with that of the NC group, significant increases in the serum insulin concentrations were noted in the HF group (Fig. 1f) in parallel with reduced insulin sensitivity in an insulin tolerance test (ITT) (Fig. 1g). The HF group showed lower high-glucose (HG)-coupled insulin secretion in a glucose-stimulated insulin secretion test (GSIS) than the NC group (Fig. 1h). *Pdx1* and *MafA*, which play an important role in pancreatic development and β cell function maintenance, decreased expression indicated dysfunction of β cells in the HF group (Fig. 1i). Overall, these data show that the offspring of mothers with an abnormal glucose metabolism present altered glucose tolerance, insulin sensitivity and insulin secretion.

In order to investigate the changes of gut microbiota in offspring and its relationship with maternal microbiota under the condition of abnormal maternal glucose metabolism, we investigated the differences in the faecal samples of the mHF group and mNC group and their respective offspring (HF and NC groups) by 16S rRNA gene amplicon sequencing. There were very few significant differences in the richness and diversity of the gut microbiota between the mHF and mNC groups during the pregnancy and lactation periods (Fig. S1a, S1b). The structure of the gut microbiota was visualized by principal coordinate analysis (PCoA), showing a distinct separation between the mHF and mNC groups, and the same result also showed in offspring groups (Fig. 1j). We also found that the gut microbiota of offspring and their mothers are closely clustered (Fig. 1j). We identified 128 amplicon sequence variants (ASVs) that contributed to the differences between the maternal mouse groups, 40 of which were depleted and 88 of which were enriched in the mHF mice compared with the mNC mice (Fig. S2). Similarly, 63 ASVs contributing to the differences between the offspring groups were identified. Compared with the NC offspring, 22 ASVs were significantly enriched, and 41 ASVs were significantly depleted in the HF offspring (Fig. 1k). Some of these ASVs in the offspring were also differentially abundant ASVs in the maternal groups, such as ASV147_Blautia enriched in both mNC group and NC offspring indicating the same changes in the microbiota observed in mothers are also present in their offspring.

In a word, we found that both HIP mothers’ glucometabolic phenotype and their gut microbiome could be ‘transmitted’ to the offspring, and dysbiosis of the gut microbiota may cause diabetes. Based on this, we speculate that the vertical transmission of the gut microbiota plays an important role in the development of the glucose metabolism status of offspring. As such, we hypothesize that blocking vertical transmission would prevent development of altered glucose metabolism in offspring of mothers with HIP.

### C-section changes glycometabolic phenotypes, microbiota composition and faecal metabolite levels in the offspring from the mice with HIP

**Fig. 2:**
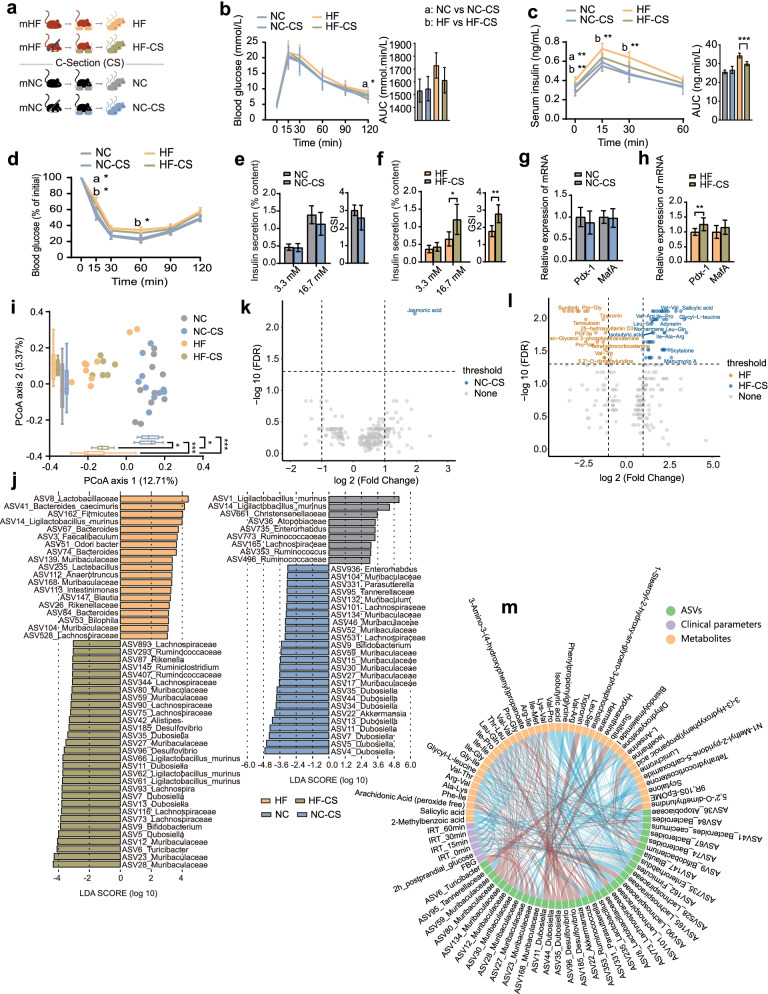
Caesarean section improved microbiota community structure, faecal metabolite levels and glycometabolic function in the offspring from mice with HIP. **a** C-section animal model and experimental design. **b**, **c** Blood glucose and serum insulin levels and AUCs during the OGTT, respectively. **d** Changes in blood glucose levels (% of initial) and AUCs during the ITT. **e**, **f** The amount of insulin secretion upon stimulation with 3.3 mmol/L (low) and 16.7 mmol/L (high) glucose and the GSI of islets isolated from pancreatic tissues of offspring of the mHF (HF and HF-CS) and offspring of the mNC (NC and NC-CS) groups, respectively. **g**, **h** Pdx-1 and MafA mRNA expression levels in pancreatic tissues of offspring of the mHF (HF and HF-CS) and offspring of the mNC (with or without CS) groups, respectively. Data are presented as the mean ± SD; *P<0.05, **P<0.01, ***P<0.001. **i** PCoA of the four offspring groups on the first two principal coordinates was performed based on the Jaccard distance. Each point represents one mouse. Comparisons were performed using the Kruskal–Wallis test for significant differences; *P<0.05, **P<0.01, ***P<0.001. **j** Identification of major ASVs contributing to the differences in the gut microbiota of offspring groups delineated by caesarean section application using LEfSe. **k**, **l** The volcano plots represent the differences in the levels of the faecal metabolites induced by Caesarean section in offspring of the mNC or mHF group. **m** Spearman’s correlation network of the offspring gut microbiota, faecal metabolites and glycometabolic profiles. The colour represents positive (red) or negative (blue) correlations. HF (*n*=10): offspring delivered by the mHF group; NC (*n*=10): offspring delivered by the mNC group; HF-CS (*n*=6): offspring born to mHF by C-section; NC-CS (*n*=10): offspring born to mNC by C-section

To investigate the effect of vertical mother-to-infant transmission of the gut microbiota via delivery on glucose metabolism, we blocked vertical microbiota transmission through C-section, and all offspring were fostered by their biological mothers (Fig. 2a).

In the offspring from the mNC group, there was no significant difference in glucose metabolism parameters as determined by an OGTT and ITT no matter whether they were born by natural delivery (NC group) or by C-section (NC-CS group) (Fig. 2b–d). For the offspring from the mHF group, although the blood glucose levels in an OGTT and AUC between the HF group (born by natural delivery) and HF-CS groups (born by C-section) were not comparable, the lower serum insulin level in the HF-CS group than the HF group showed an altered insulin resistance of the offspring derived from the mHF group was mitigated by C-section (Fig. 2b–d). GSIS test results and Pdx-1 mRNA expression also indicated improved glucose metabolism in C-section-delivered offspring from the mHF group (Fig. 2e–h). Given these results, we can conclude that C-section alleviates the impaired islet β cell secretion function and insulin resistance of the offspring from HIP mice.

The microbial diversity of the NC-CS group was significantly higher than that of the NC group, whereas for the offspring from the mHF group the microbial diversity of the HF-CS group showed no difference from that of the HF group (Fig. S3). The linear discriminant analysis effect size (LEfSe) analysis of the relative abundance of individual ASVs showed that 9 ASVs had a significantly lower relative abundance and 37 ASVs had a higher relative abundance in the NC-CS group compared with those in the NC group (Fig. 2j). For the offspring of the mHF group, C-section resulted in a significantly lower relative abundance of 19 ASVs and enrichment of 30 ASVs (Fig. 2j). Compared with vaginal delivery groups (NC and HF group), the relative abundances of ASVs classified into the family Muribaculaceae and genus *Dubosiella* were significantly higher in the C-section delivery groups (NC-CS and HF-CS group). By contrast, the relative abundances of ASVs classified into the genus *Bacteroides* were decreased, and those of ASVs classified into the family Lachnospiraceae were increased with C-section only in the offspring of the mHF group (HF-CS group). Interestingly, the relative abundances of ASVs classified into the family Ruminococcaceae were lower in the HF-CS group than in the HF group but higher in the NC-CS group than in the NC group.

Since microbiota metabolites are an important way for the gut microbiota to function, untargeted metabolome profiles were generated on faecal samples by LC-MS. From an orthogonal partial least squares discriminant analysis (OPLS-DA) model, we identified two sets of differentially produced compounds that included 228 metabolites under positive and negative ion modes in the offspring of the mHF group and 222 metabolites in the offspring of the mNC group (Fig. S5). Compared with natural delivered offspring from the mHF group, dipeptides containing branched-chain amino acids (BCAAs) (valine, leucine, isoleucine), salicylic acid and isobutyric acid were more abundant, while dipeptides containing proline were less abundant, in the C-section delivered offspring from mHF (HF-CS group versus HF group) (Fig. 2l). Of note, for offspring from the mNC group, only jasmonic acid showed differentially abundant metabolites in the NC-CS versus NC groups (Fig. 2k).

Subsequently, we analysed Spearman’s correlation network of the offspring’s gut microbiota, faecal metabolites and glycometabolic profiles to further explore the characteristics of the microbiota in glucose metabolism (Fig. 2m). This analysis identified positive correlations between the relative abundances of most ASVs of Muribaculaceae and blood glucose and serum insulin levels and negative correlations between the relative abundances of most ASVs of Ruminococcaceae and serum insulin levels. In addition, the relative abundances of dipeptides containing BCAAs (valine, leucine and isoleucine) were markedly negatively correlated with serum insulin levels; however, the relative abundances of dipeptides containing proline showed an opposite correlation with glucose levels. The relative abundances of some ASVs of Muribaculaceae and Ruminococcaceae were positively correlated with the relative abundances of dipeptides containing BCAAs.

In summary, after blocking microbial vertical transmission by C-section, insulin resistance was ameliorated and insulin secretion was promoted in the offspring of mice with HFD-induced impairment of glucose metabolism, and these changes were accompanied by changes in faecal metabolite levels.

### Microbiota transmission by cross-fostering causes the microbiota composition and glycometabolic phenotype of offspring to trend toward those of their foster mother

**Fig. 3:**
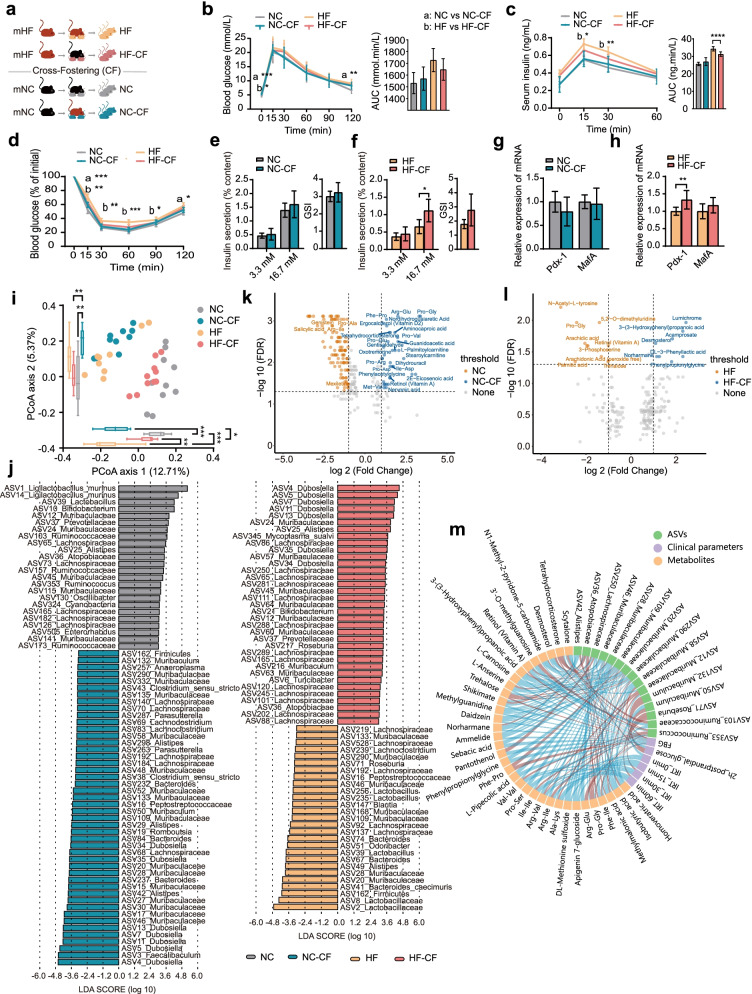
Alteration of microbiota transmission by cross-fostering causes the gut microbiota composition and glycometabolic phenotype of offspring to trend toward those of their foster mother. **a** Cross-fostering animal model and experimental design. **b**, **c** Blood glucose and serum insulin levels and AUCs during the OGTT, respectively. **d** Changes in blood glucose levels (% of initial) and AUCs during the ITT. **e**, **f** The amount of insulin secretion upon stimulation with 3.3 mmol/L (low) and 16.7 mmol/L (high) glucose and the GSI of islets isolated from pancreatic tissues of offspring of the mHF (HF and HF-CF) and offspring of the mNC (NC and NC-CF) groups, respectively. **g**, **h** Pdx-1 and MafA mRNA expression levels in pancreatic tissues of offspring of the mHF (HF and HF-CF) and offspring of the mNC (NC and NC-CF) groups, respectively. Data are presented as the mean ± SD; *P<0.05, **P<0.01, ***P<0.001. **i** Principal coordinate analysis (PCoA) of the four offspring groups on the first two principal coordinates was performed based on the Jaccard distance. Each point represents one mouse. Comparisons were performed using the Kruskal–Wallis test for significant differences; *P<0.05, **P<0.01, ***P<0.001. **j** Identification of major amplicon sequence variants (ASVs) contributing to the differences in the gut microbiota of offspring groups delineated by CF using LEfSe. **k**, **l** Volcano plots represent the differences in faecal metabolite levels induced by CF in offspring of the mNC or mHF group. **m** Spearman’s correlation network of the offspring gut microbiota, faecal metabolites and glycometabolic profiles. The colour represents positive (red) or negative (blue) correlations. HF (*n*=10): offspring delivered by the mHF group; NC (*n*=10): offspring delivered by the mNC group; HF-CF (*n*=10): offspring delivered by the mHF group and cross-fostered by the mNC group; NC-CF (*n*=10): offspring delivered by mNC and cross-fostered by the mHF group

We further evaluated the effect of vertical transmission of the gut microbiota through nursing on glucose metabolism by implementing cross-fostering in our experimental model. After natural delivery, offspring were fostered by the mother from the other group (e.g. offspring born from the mNC group but fostered by the mHF group named NC-CF) (Fig. 3a). In the offspring from the mNC group, although there were no differences in glucose tolerance and insulin resistance between the NC-CF and NC groups (Fig. 3b, c), insulin sensitivity of NC-CF group showed an impairment (Fig. 3d). Nevertheless, for the offspring from the mHF group compared with the HF group, cross-fostering was also to relieve hyperinsulinism (Fig. 3c), accelerate insulin sensitivity (Fig. 3d) and potentiate islet function (Fig. 3f, h) in the HF-CF group. These results indicate that cross-fostering ameliorated the impaired glycometabolic phenotype of HF offspring.

As for gut microbiota, the α-diversity of the NC-CF group was significantly higher than that of the NC group (Fig. S3). PCoA results suggested that offspring fostered by mothers from the same group had a similar gut microbiota structure (Fig. 3i). An analysis of major ASVs contributing to the differences among groups showed that 70 ASVs (24 ASVs depleted and 46 ASVs enriched) were significantly differentially abundant between the NC and NC-CF groups, and 62 ASVs (27 ASVs depleted and 35 ASVs enriched) were significantly differentially abundant between the HF and HF-CF groups (Fig. 3j). The number of differentially abundant ASVs induced by cross-fostering was greater than that induced by C-section, indicating that maternal nursing had more profound effect on the establishment of gut microbiota of offspring than delivery mode. Although ASVs classified into the family Muribaculaceae and family Lachnospiraceae showed bidirectional changes in relative abundance in all offspring groups, the relative abundances of most ASVs of Muribaculaceae increased in the NC-CF group, and those of most ASVs of Lachnospiraceae increased in the HF-CF group. The relative abundances of Bacteroides and Alistipes were also higher in the NC-CF group than in the NC group, while those of Bifidobacterium and Ruminococcaceae were lower in the NC-CF group than in the NC group. Among the offspring of the mHF group mothers, the relative abundance of ASVs classified as Lactobacillus and Bacteroides (including *Bacteroides caecimuris*) decreased in the HF-CF group (Fig. 3j).

Regarding faecal metabolites of offspring groups (NC-CF group, NC group, HF-CF group and HF group), we identified 325 metabolites under positive and negative ion modes in the offspring of the mNC group mothers from the OPLS-DA model and 206 metabolites in the offspring of the mHF group mothers (Fig. S6). The levels of dipeptides containing proline, fat-soluble vitamins (e.g. ergocalciferol and retinol) and aminocaproic acid were higher in the NC-CF group than in the NC group, whereas salicylic acid, phenylpropanoid and polyketide (e.g. genistein) levels were lower (Fig. 3k). Compared with those in the HF group, we found that 3-(3-hydroxyphenyl) propanoic acid and lump chrome levels were higher in the HF-CF group, while the levels of N-acetyl-L-tyrosine, lipids and lipid-like molecules (e.g. retinol and arachidonic acid) and palmitic acid were lower in the HF-CF group (Fig. 3l).

Correlation analysis revealed that the relative abundances of most ASVs classified into the family Muribaculaceae were positively correlated with blood glucose levels and serum insulin levels. Nevertheless, the relative abundances of only one ASV of Atopobiaceae and two ASVs of Ruminococcaceae were negatively correlated with fasting blood glucose (FBG) and 2 h postprandial glucose (2h-PG) levels, respectively. Notably, the relative abundances of dipeptides containing proline, lipids and lipid-like molecules (e.g. retinol and arachidonic acid) were positively correlated with blood glucose levels, and the relative abundances of dipeptides containing isoleucine were negatively correlated with serum insulin levels. The relative abundances of most ASVs of Muribaculaceae showed a positive correlation with the relative abundances of dipeptides containing proline and a negative correlation with the relative abundances of genistein and dipeptides containing proline. The relative abundances of ASVs of Ruminococcaceae and Atopobiaceae showed a positive correlation with the relative abundances of dipeptides containing isoleucine (Fig. 3m).

Altogether, these results indicate that microbiota transmission via nursing made the microbiota composition of offspring trend toward that of their foster mother; this phenomenon is also observed with the glycometabolic phenotype.

### The combination of C-section and cross-fostering showed more significant effects on the gut microbiota and glycometabolic phenotypes of the offspring from the mHF group mice

**Fig. 4:**
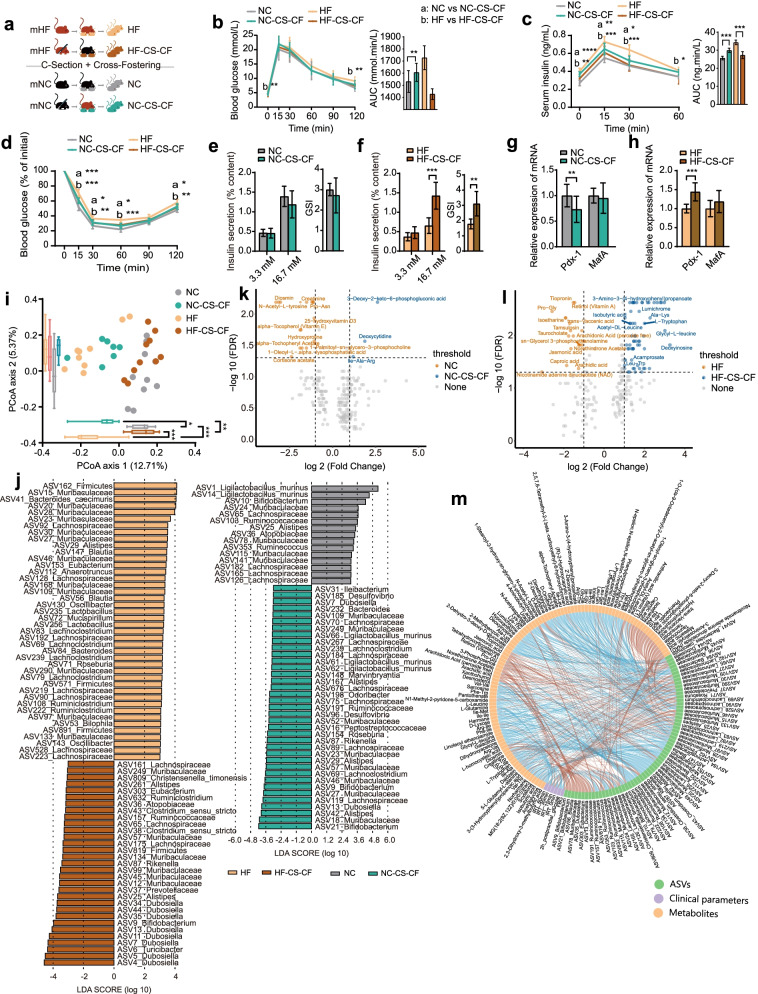
The combination of C-section and cross-fostering showed more significant effects on gut microbiota and glycometabolic phenotypes in the offspring of the mHF group. **a** C-section and cross-fostering animal model and experimental design. **b**, **c** Curves of blood glucose levels and serum insulin levels during the OGTT and AUC, respectively. **d** Changes in blood glucose levels (% of initial) during the ITT and AUC. **e**, **f** The amount of insulin secretion at 3.3 mmol/L (low) and 16.7 mmol/L (high) glucose and GSI of islets isolated from pancreatic tissues of offspring of the mHF (HF and HF-CS-CF) and offspring of the mNC (NC and NC-CS-CF) groups, respectively. **g**, **h** Pdx-1 and MafA mRNA expression levels in pancreatic tissues of offspring of the mHF (HF and HF-CS-CF) and mNC (NC and NC-CS-CF) groups, respectively. Data are presented as the mean ± SD; *P<0.05, **P<0.01, ***P<0.001. **i** Principal coordinate analysis (PCoA) of the four offspring groups on the first two principal coordinates was performed based on the Jaccard distance. Comparisons were performed using the Kruskal–Wallis test for significant differences; *P<0.05, **P<0.01, ***P<0.001. **j** Identification of major amplicon sequence variants (ASVs) contributing to the differences in the gut microbiota of offspring groups by C-section combined with cross-fostering using LEfSe. **k**, **l** Volcano plots represent the differences in offspring faecal metabolites induced by C-section combined with cross-fostering in offspring of the mNC or mHF group. **m** Spearman’s correlation network of the gut microbiota, faecal metabolites and glycometabolic profiles in the context of offspring. The colour represents positive (red) or negative (blue) correlations. HF (*n*=10): offspring delivered by the mHF group; NC (*n*=10): offspring delivered by the mNC group. HF-CS-CF (*n*=7); offspring born to mHF by C-section and cross-fostered by the mNC group; NC-CS-CF (*n*=10): offspring born to mNC by C-section and cross-fostered by the mHF group

Given the role of delivery mode and maternal nursing in shaping the gut microbiota, the effect to block microbiota transmission via both mechanisms on glucose metabolism of the offspring was studied. To this end, we subjected offspring of two maternal groups to both delivery by C-section and cross-fostering (HF-CS-CF and NC-CS-CF, respectively) and compared glucose metabolism and microbiome with those of natural delivery and maternal nursing (HF and NC, respectively) (Fig. 4a). Compared with the NC group, the offspring from mNC that was delivered by C-section and underwent mHF fostering (the NC-CS-CF group) exhibited impaired insulin sensitivity (Fig. 4d), and serum insulin levels in the NC-CS-CF group were higher than those in the NC-CF group (*p*<0.05) (Fig. S4), suggesting that the effect of the combination of the two blocking methods was stronger than that of either individually. Otherwise, in the offspring from mHF, we observed that glucose intolerance and insulin resistance were mitigated in the HF-CS-CF group compared with the HF group (Fig. 4b–d). More importantly, both the blood glucose levels and serum insulin levels in the HF-CS-CF group were lower than those in the HF-CF group (Fig. S4), suggesting that C-section amplified the effect of cross-fostering. Additionally, C-section combined with mNC fostering strengthened HG-coupled insulin secretion in the HF offspring (Fig. 4f). A similar tendency could be observed for the expression of Pdx-1 (Fig. 4h). However, the results of the NC-CS-CF group versus the NC group showed no statistical difference (Fig. 4e, g).

The structure of the gut microbiota in offspring differed significantly after C-section and cross-fostering compared to the NC and HF groups respectively (Fig. 4i). Additionally, the structure of their microbiota was aligned with that of their foster mother (Fig. 4i). There were 74 ASVs (42 ASVs depleted and 32 enriched) that were significantly differentially abundant between the HF-CS-CF and HF groups, and there were 52 ASVs (15 ASVs depleted and 37 enriched) that were significantly differentially abundant between the NC-CS-CF and NC groups (Fig. 4j). Compared to the NC-CS-CF and HF-CS-CF groups, respectively, linear discriminant analysis effect size (LEfSe) of the three blocking methods revealed that the relative abundances of several bacteria showed similar changes in both the NC and HF groups; compared with the NC group, the relative abundances of ASVs classified into *Ligilactobacillus murinus*, ASV165_Lachnospiraceae and ASV353_Ruminococcus were decreased in the other offspring of the mNC group (NC-CS, NC-CF and NC-CS-CF group). And compared with the HF group, those of ASV41_Bacteroides_caecimuris, ASV162_Firmicutes, ASV147_Blautia, etc., were decreased in the other offspring of the mHF group (HF-CS, HF-CF and HF-CS-CF groups) (Fig. 4l).

From the OPLS-DA model, we identified two collections of differentially produced compounds that included 212 metabolites under positive and negative ion modes in the offspring of the mHF group and 222 metabolites in the offspring of the mNC group (Fig. S7). Few metabolites showed higher levels in the NC-CS-CF group than in the HF group, while lipids and lipid-like molecules (e.g. 25-hydroxyvitamin D3) showed lower levels (Fig. 4k). The levels of most organic acids and derivatives (e.g. glycyl-L-leucine) were higher in the HF-CS-CF group than in the HF group. As found in the comparison of the HF-CS group and the HF group, the level of isobutyric acid was higher in the HF-CS-CF group than in the HF group; moreover, as found in the comparison of the HF-CF group and the HF group, the levels of lipids and lipid-like molecules were lower in the HF-CS-CF group than in the HF group (Fig. 4l).

Finally, correlation analysis showed that most ASVs of Lachnoclostridium were positively correlated but most ASVs of Ruminococcaceae were negatively correlated with blood glucose levels and serum insulin levels (Fig. 4m). ASVs of Lachnospiraceae and Muribaculaceae showed a dual correlation with the gut microbiota and glycometabolic phenotypes. Most faecal metabolites were negatively correlated with metabolic parameters, including salicylic acid, while dipeptides containing proline, lipids and lipid-like molecules (e.g. retinol, arachidonic acid and 3-phosphoserine) showed the opposite correlations with faecal metabolites, as observed with the groups with only cross-fostering intervention. Salicylic acid was negatively associated with ASV162_Firmicutes.

Collectively, the results suggested that the combination of C-section and cross-fostering showed more significant effects than either intervention alone on the glycometabolic phenotypes of the offspring of both the mNC and mHF groups, and marked differences in the gut microbiota structure and faecal metabolite levels were observed only in the offspring of the mHF group.

### C-section or cross-fostering affects the vertical transmission of the gut microbiota

**Fig. 5:**
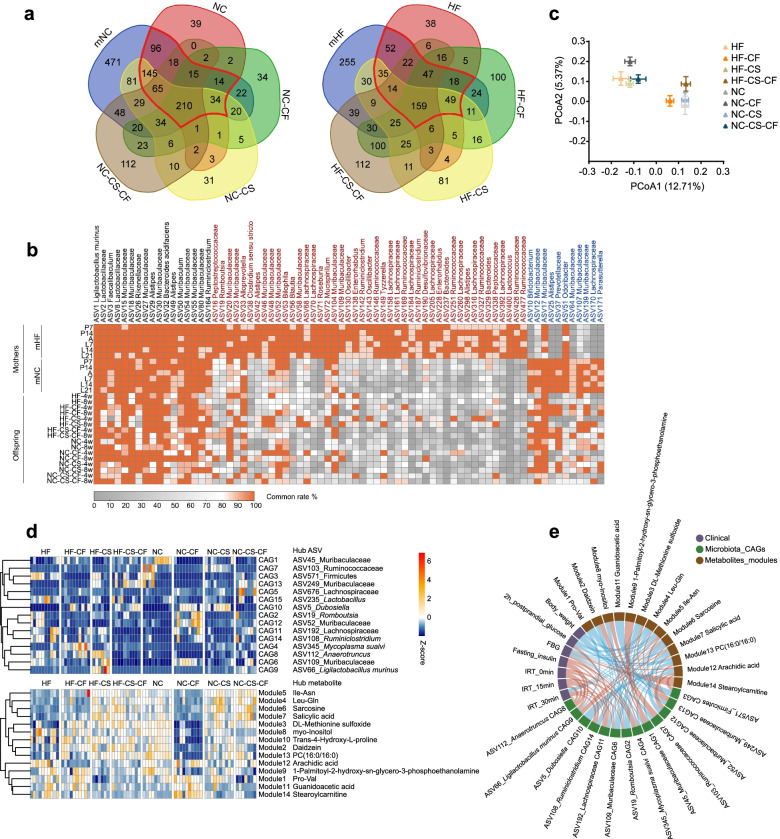
C-section and cross-fostering both interrupt the vertical transmission of the gut microbiota. **a** Venn diagrams showing that mothers with different glucose metabolism profiles share ASVs with their offspring. **b** Heatmap showing the maternal core gut microbiota in the different mother groups defined according to the common rate of over 80%, demonstrating the vertical transmission of the maternal core gut microbiota. The ASVs labelled with black text represent part of the core microbiota of both high-fat diet-fed and normal chow diet-fed mothers, the ASVs labelled with red text are part of the core microbiota of high-fat diet-fed mothers only and the ASVs labelled with blue text are part of the core microbiota of normal chow diet-fed mothers only. **c** Principal coordinate analysis (PCoA) of all offspring groups on the first two principal coordinates was performed based on the Jaccard distance. **d** The relative abundance of co-abundant groups (CAGs) of microbiota ASVs in offspring mice and the relative abundance of co-abundant modules of faecal metabolites in offspring mice. **e** The spearman’s correlation network of microbiota CAGs, faecal metabolites modules and clinical indexes. The colour represents positive (red) or negative (blue) correlations. The ASV or metabolite labelled beside correspond CAG or module was the hub ASV or metabolite. mHF (*n*=7): maternal group with high-fat diet; mNC (*n*=8): maternal group with normal control diet; HF (*n*=10): offspring delivered by the mHF group; NC (*n*=10): offspring delivered by the mNC group. HF-CS (*n*=6): offspring born to mHF by C-section; NC-CS (*n*=10): offspring born to mNC by C-section. HF-CF (*n*=10): offspring delivered by the mHF group and cross-fostered by the mNC group; NC-CF (*n*=10): offspring delivered by mNC and cross-fostered by the mHF group. HF-CS-CF (*n*=7); offspring born to mHF by C-section and cross-fostered by the mNC group; NC-CS-CF (*n*=10): offspring born to mNC by C-section and cross-fostered by the mHF group

To explore the vertical transmission of the gut microbiota under the different glucose metabolism statuses, we explored the shared microbial taxa between birth mothers and their offspring independently of the delivery method and foster mother. For the mNC group and their offspring, 597 (96+145+65+18+210+15+34+14) shared ASVs were observed between the mNC group and NC offspring group, and 618 and 369 shared ASVs were found between the mNC group and the NC-CS and NC-CF groups, respectively (Fig. 5a). Additionally, 373 ASVs were transmitted from the mNC group mothers to the NC-CS-CF group offspring. Among the 597 shared ASVs between the mNC and NC groups, the transmission of 143 (96+18+15+14) ASVs to the offspring was blocked by C-section, and that of 328 (145+96+65+18) ASVs was blocked by cross-fostering. The combination of C-section and cross-fostering blocked the transmission of 289 (145+96+34+14) shared ASVs. The mHF group and HF offspring groups had 396 shared ASVs (52+35+22+14+47+159+18+49), and there were 332 and 363 shared ASVs between the mHF group and the HF-CS and HF-CF groups, respectively. In addition, 345 ASVs were transmitted to the HF-CS-CF group from the mHF group. Among the 396 shared ASVs, the transmission of 139 (52+22+47+18) ASVs to offspring was blocked by C-section, and that of 123 (52+35+14+22) ASVs was blocked by cross-fostering. The combination of C-section and cross-fostering blocked the transmission of 154 (52+35+18+49) shared ASVs.

The shared gut microbiota was defined according to the common rate of over 80% to show the vertical transmission of the maternal core gut microbiota. A heatmap showed that most of the ASVs shared by the mHF and mNC groups were transmitted to the offspring. Interestingly, most ASVs specific to the mNC group were vertically transmitted to the offspring, but ASVs specific to the mHF group were rarely transmitted to the offspring. Additionally, cross-fostering significantly increased the common rate of some core microbiota constituents specific to the lactating mother in the offspring. The above phenomenon in the single blocking method could be observed in the C-section combined with cross-fostering groups (Fig. 5b).

We further did an integrative analysis of gut microbiota from all offspring groups. Besides different structures of gut microbiota in offspring (C-section, cross-fostering and cross-fostering immediately after C-section respectively) mentioned above (Figs. 2i, 3i, and 4i), we found some interesting gut microbiota characteristics of offspring relevant with the microbiota transmission from mother to offspring via nursing. The two offspring groups (HF-CF and HF-CS-CF) born from the mHF group and fostered by the mNC group showed similar structure of gut microbiota of the other two offspring groups (NC and NC-CS) born from the mNC group, no matter they were born by natural delivery or by C-section, and similar results were found in offspring born from the mNC group and fostered by the mHF group. It indicates that cross-fostering exert a profound influence on the gut microbiota of offspring than C-section (Fig. 5c).

All the different ASVs identified between offspring groups in the above analysis were clustered into 15 co-abundance groups (CAGs) based on their SparCC correlations (Fig. 5d). There are 3 ASVs (ASV19_Romboutsia, ASV52_Muribaculaceae and ASV109_Muribaculaceae) not only the hub ASV of 3 CAGs (CAG2, CAG12 and CAG109, respectively), but also the members of the maternal core ASVs specific in the mHF group (Fig. 5b, d). All the differential metabolites between offspring groups in the above analysis were clustered into 12 modules based on their bicor correlations (Fig. 5d). Based on this, we analysed the correlations among the CAGs, faecal metabolite modules and glucometabolic phenotypes in all offspring groups (Fig. 5e). Interestingly, the 2 CAGs we mentioned above, CAG2 and CAG6, were both positively correlated with two glucose metabolism-related parameters (FBG and IRT_30min) and negatively correlated with six faecal metabolite modules (module2, 3, 4, 6, 7, 13) including dipeptide-containing leucine (a BCAA) and salicylic acid, and these faecal metabolite modules showed positive correlations with the two glucose metabolism-related parameters (FBG and IRT_30min) mentioned above.

## Discussion

In this study, we found that vertical transmission of the specific bacteria and their metabolites contributed to the development of glucose metabolism defects in the offspring from mothers with HIP. Furthermore, the similarity of gut microbial structure between offspring and their fostering mothers was higher than that between children and their birth mothers.

In humans, infants exposed to maternal hyperglycaemia during the prenatal period have increased risks of obesity and insulin resistance in childhood and impaired glucose tolerance and T2DM in adulthood [19–22]. Likewise, our data confirmed that the offspring of mice with HFD mothers presented impaired glucose tolerance, insulin sensitivity and insulin secretion, which was consistent with the previous animal study [11]. Furthermore, substantial changes to the structure and composition of the gut microbiota were observed in HFD-induced fed maternal mice and their offspring, and shared microbial strains were observed between generations. On the basis that gut microbiota has been proved as an initiating factor and an intervention target for T2DM [23], we reasonably inferred that the vertical transmission of the gut microbiota promoted the inheritance of glucose metabolism deficits from mother to offspring.

The maternal microbiota is the first source for the establishment of the gut microbiota in offspring, and this colonization occurs during intrauterine development, delivery and postnatal feeding [24, 25]. It has long been thought that infants live in a sterile environment before birth, but some studies have shown that gut microbiota colonization of infants may occur before birth [26]. It remains controversial about the establishment of gut microbiota in the uterus. In this study, we mainly focused on the microbiota transmission during production and postpartum, of which delivery mode and fostering are the key processes.

To verify the hypothesis that vertical transmission of the gut microbiota plays a role in the inheritance of glucose metabolism deficits from mother to offspring, we blocked microbial transmission through C-section and cross-fostering. C-section is reported to increase the risk of obesity in childhood by 46% in healthy pregnancy [27]. However, in this study, C-section marginally improved the insulin resistance of the offspring of the mHF group and partially rescued the secretion function of islet β cells. We reasoned that the improved insulin resistance and islet β cell secretion function were related to the decrease in relative abundance of Muribaculaceae and the increase in the relative abundance of Ruminococcaceae during the vertical transmission of the gut microbiota. A study based on an animal model of T2DM reported that acarbose rescues the decrease in Muribaculaceae relative abundance while lowering blood glucose [28]. Interestingly, some studies support our conclusion that Ruminococcaceae is a contributor to the hypoglycaemic effect; however, fewer studies have reported positive associations of Ruminococcaceae with hyperglycaemia [29].

Another possible reason for the improvement of glucose metabolism with C-section delivery of offspring from the mice with HIP may be attributed to the gut microbiota metabolites. For example, C-section significantly increased the level of isobutyric acid, which induces browning of white fat and hepatic oxidation via PPARα, in the offspring of mHF mice [30]. BCAA was another class of metabolites that are significantly altered after blocking microbiota transmission. Specifically, BCAA relative abundances were increased in the mHF offspring mice with C-section, and they were negatively correlated with blood glucose and serum insulin levels. However, many studies have defined elevated serum BCAA levels as a biomarker for the development of metabolic syndrome [31]. A possible explanation is a difference between gut-derived BCAAs and circulating BCAAs, which is supported by animal study results showing that a diet specifically enriched in leucine (a BCAA) could improve glucose homeostasis [32]. Further investigations are needed to better understand the relationship between different features of the gut microbiota and host- or microbially derived metabolites in the blood. On the basis of the effect of C-section, it is potential for the newborns from HIP mothers to depress the risk of glucose metabolism deficits.

After birth, maternal milk is the primary factor that shapes the gut microbiota of offspring [33]. In the current work, mHF cross-fostering increased Lactobacillus relative abundance in the mice with NC birth mothers, which is a component of the core microbiota in breast milk during lactation [34, 35]. Our results suggested that Lactobacillus was likely to be transmitted via the milk of the mHF group to pups being nursed. Moreover, we concluded from the results that the gut microbial structure of the offspring shifted to that of the corresponding foster mother, as it contained ASVs unique to the foster mother. Similar to our study, a prospective, longitudinal study also reported that an infant’s gut microbial communities were more closely related to its mother’s milk and skin than to those of a random mother [36]. It is novel to discover that the effect of fostering was more profound than that of C-section for the establishment of the gut microbiota, supported by the greater number of differentially abundant ASVs associated with cross-fostering than with C-section in this study. Most recently, a case-control study designed to examine type I diabetes in children also regarded fostering as the most significant factor associated with microbiota composition [37]. It deserves more discussion on the use of cross-fostering to treat gut microbiota-related diseases.

While there is no study that identified the specific microbial species and its metabolites whose relative abundance significantly changes between offspring that are cross-fostered, our design allows us to observe that mHF fostering increased Bacteroidaceae relative abundance but decreased Bifidobacterium relative abundance in the mice with NC birth mothers, and mNC fostering correspondingly decreased Bifidobacterium relative abundance in the mice with HF birth mothers. Previous studies on the effect of the genus Bacteroides on glucose metabolism have reached inconsistent conclusions. Several cross-sectional studies have noted a negative association between the relative abundance of Bacteroides and the incidence of T2DM [29], but several clinical studies reported that the ameliorative effect of metformin treatment or bariatric surgery on diabetes was related to a decrease in Bacteroidaceae relative abundance [38, 39]. The increase in Bacteroides relative abundance induced by mHF fostering in our study may be explained by the potential compensatory mechanisms by which the gut microbiota contributes to impaired metabolism function. On the other hand, mNC fostering decreased the level of palmitic acid, a known stimulator of the proinflammatory response and insulin resistance [40, 41], in the mice with HF birth mother. It was another explanation of mNC cross-fostering improving insulin sensitivity in offspring from the mHF group. The protective effect of high Bifidobacterium relative abundance against diabetes is widely accepted. Extensive clinical and animal studies have highlighted the ability of Bifidobacterium to lower fat mass, increase insulin sensitivity and improve inflammation by producing specific SCFAs. Extensive clinical and animal studies have highlighted the ability of Bifidobacterium to lower fat mass, increase insulin sensitivity and improve inflammation by producing specific SCFAs [42, 43]. Maternal and infant studies have shown that breastfeeding is associated with higher levels of bifidobacterium [37]. Comprehensive analysis of infant cohort data from different regions showed that Bifidobacterium deficiency is significantly associated with gene deficiency related to breast milk utilization, which may have adverse consequences on immunity in the long term [44]. Therefore, we reason that the Bifidobacterium relative abundance resulting from gut microbiota transmission plays a critical effect on glucose metabolism. These key factors of gut microbiota and metabolites affecting glucose metabolism of offspring need further studies in validating the causal relationship and specific mechanism between them and glucose metabolism of offspring. Bacteria and metabolites that truly regulate glucose metabolism of offspring may be used as new microbiome targeting therapies in the clinic.

In this study, we also explore the cross-talk between delivery method and fostering by NC-CS-CF and HF-CS-CF groups. mHF fostering influence on the faecal metabolites of their offspring is very remarkable through vaginal delivery no matter who the birth mothers were, but which was almost completely suppressed when C-section. Nevertheless, the influence of mNC fostering was hardly affected by delivery mode, and even expressed a stronger influence with C-section. These results suggested that delivery mode and fostering did not act independently in the vertical transmission of gut microbiota, but their effects of the “inherence” of impaired glucose metabolism are far from superimposed.

## Conclusion

Partially blocking vertical microbial transmission led to improvement in insulin sensitivity and islet function of the offspring. Importantly, microbiota acquired via birth and nursing respectively are significantly different and they play different roles in the formation of the structure and composition of the gut microbiota and faecal metabolites.

## Materials and methods

### Animal care

Specific pathogen-free (SPF) C57BL/6 mice at 7 weeks of age were purchased from Beijing Vital River Laboratory Animal Technology Co., Ltd. (Beijing, China, SCXK-2016-0006) and housed in a standard SPF environment (room temperature, 21-25 °C; room humidity, 30–70%; 12-h light/12-h dark cycle) with free access to food and water. All protocols were approved by the Institutional Animal Care and Use Committee (IACUC) of Renmin Hospital of Zhengzhou University.

### Study design

After 1 week of adaptive feeding, female mice were randomly assigned to two groups, and they were given a HFD (containing 60% kcal fat, Research Diets, D12492) or a NCD for 8 weeks. After 8 weeks of HFD induction, an oral OGTT was performed to evaluate the degree of insulin resistance in the female mice. Subsequently, female mice mated with male mice, and all litters were weaned at 3 weeks and fed a NCD until 8 weeks of age.

The presence of a vaginal plug was marked as gestational day 0.5 (G0.5), and female mice were euthanized by cervical dislocation at gestational day 19.5 (G19.5). The abdominal skin was prepared by application of 70% ethanol before incision, and the uterus was removed and placed on sterile gauze. Pups in litters born within 12 h of each other were subjected to cross-fostering. After birth, the litters were placed with their respective foster mothers (HFD- or NCD-fed mice) until weaning.

All offspring in our study were divided into 8 groups, which were (1) HF, born to HFD-fed mothers (mHF) by vaginal delivery and fostered by the mHF group; (2) HF-CS, born to the mHF group by C-section and fostered by the mHF group; (3) HF-CF, born to the mHF group by vaginal delivery and cross-fostered by NCD-fed mothers (mNC group); (4) HF-CS-CF, born to the mHF group by C-section and cross-fostered by the mNC group; (5) NC, born to the mNC group by vaginal delivery and fostered by the mNC group; (6) NC-CS, born to the mNC group by C-section and fostered by the mNC group; (7) NC-CF, born to the mNC group by vaginal delivery and cross-fostered by the mHF group; and (8) NC-CS-CF, born to the mNC group by C-section and cross-fostered by the mHF group. The pancreas was collected from all offspring at 8 weeks, and faecal samples were collected at both 4 and 8 weeks.

### OGTT and ITT

When the offspring were 4 weeks old, an OGTT was performed by orogastric gavage of 50% glucose (2 g/kg body weight) after overnight fasting [45]. Blood (5–10 μL) was collected from the tail vein at 0, 15, 30, 60, 90 and 120 min following glucose administration to measure blood glucose by glucometer. Serum was collected from the blood by centrifugation at 3000 rpm for 15 min, and the serum insulin concentration was quantified with an ELISA kit (Crystal Chem Inc., Downers Grove, IL). An ITT was performed by intraperitoneal injection of recombinant human short-acting insulin (0.75 U/kg) after 4 h of fasting. Blood (5–10 μL) was collected at 0, 15, 30, 60 and 90 min following insulin administration for blood glucose measurement.

### Isolation and culture of primary islet cells

Upon euthanasia, the pancreas and bile duct were exposed to air. A bile duct catheter was introduced, followed by digestion via injection of collagenase (Sigma, USA). Then, pancreatic islets were isolated and collected individually under a microscope. Islets were cultured overnight with RPMI-1640 (GIBCO, USA) medium at 37 °C in a humidified 5% CO_2_ atmosphere. Subsequently, islets were washed twice with a buffer solution (pH 7.4) containing 20 mmol/L HEPES, 115 mmol/L NaCl, 5 mmol/L NaHCO_3_, 5 mmol/L KCl, 2.6 mmol/L CaCl_2_, 1.2 mmol/L KH_2_PO_4_, 1.2 mmol/L MgSO_4_, 3 mmol/L D-glucose and 1% (w/v) bovine serum albumin (BSA) [46].

### GSIS

A GSIS was performed as previously described. Briefly, cultured primary islet cells were preincubated with 200 μL of glucose-free KRBH buffer containing 675 mmol/L NaCl, 18 mmol/L KCl, 2.5 mmol/L MgSO4·7H2O, 2.5 mmol/L NaH2PO4·2H2O, 7.5 mmol/L CaCl2·2H2O, 10 mmol/L NaHCO3, 50 mmol/L HEPES and 1% BSA for 1 h at 37 °C. Then, the cells were exposed to 200 μL of KRBH buffer containing 3.3 mmol/L or 16.7 mmol/L glucose for a 1-h incubation at 37 °C. The insulin content of the supernatant was quantified with a mouse insulin ELISA kit (Crystal Chem, USA). Glucose stimulation index (GSI) = insulin release in high glucose state/insulin release in low glucose state.

### Quantitative real-time PCR (qPCR)

Total RNA was extracted from the pancreas using TRIzol reagent. First-strand cDNA was synthesized using a RevertAid First-Strand cDNA Synthesis Kit (Thermo Fisher Scientific, USA), and qPCR assays were performed using SYBR Green qPCR Master Mix on a Roche LightCycler 480 II Sequence Detection System (Roche Diagnostics). β-actin served as the reference gene, and the primer sequences were as follows: Pdx-1, F: 5′-ATGAACAGTGAGGAGCAGTACTACG-3′ and R: 5′-GGAGCCCAGGTTGTCTAAAT-3′; MafA, F: 5′-ATCATCACTCTGCCCACCAT-3′ and R: 5′-AGTCGGATGACCTCCTCCTT-3′; and β-actin, F: 5′-TGACAGGATGCAGAAGGAGA-3′ and R: 5′-GCTGGAAGGTGGACAGTGAG-3′.

### Statistical analysis

All data are presented as the mean±SD. Two-tailed independent Student’s *t* test was used to assess the significance of differences between two groups. One-way analysis of variance (ANOVA) was used for comparisons among three or more groups, followed by the Holm–Šidák or Tukey post hoc test for all pairwise multiple comparisons. A *P* value <0.05 was considered indicative of significance.

### Faecal bacterial community determination

Faeces from maternal mice were collected at day 7 and day 14 during pregnancy and antepartum and day 7 and day 14 during lactation. Faeces from offspring were collected at 4 weeks and 8 weeks of age. Faecal samples from the progenies of the two groups were taken at the age of 4 weeks and 8 weeks, respectively, and immediately placed in a −80°C refrigerator or liquid nitrogen for long-term storage. Avoid urine contamination of stool samples during collection. All samples were sequenced and analysed after collection.

Genomic DNA was extracted from frozen faecal samples using a QIAamp PowerFecal Pro DNA Kit (QIAGEN, USA, 51804) according to the manufacturer’s instructions. For 16S rRNA gene amplicon sequencing, polymerase chain reaction (PCR) targeting the V3-V4 region of the 16S rRNA gene was performed with the following primers: 5′-CCTACGGGNGGCWGCAG-3′ (forward) and 5′-GACTACHVGGGTATCTAATCC-3′ (reverse) [47]. 16S rRNA amplicon library was prepared by two steps PCR. There are 21 cycles in PCR for V3-V4 region 16S rRNA gene and 8 cycles in index PCR. Subsequent amplicon sequencing was performed on the MiSeq platform (Illumina, USA) to generate 300-bp paired-end reads. Sequencing data were demultiplexed using the QIIME2 pipeline, version 2019.7. For all the sequencing data, forward reads were trimmed at 261 bp, and reverse reads were trimmed at 229 bp. An average of 51,869 reads were used as input, with an average of 32,730 reads recovered after filtering, denoising, merging forward and reverse reads and removing chimaeras. ASVs were identified with the DADA2 plugin [48]. All the samples were randomly subsampled to equal depths of 14016 reads prior to the subsequent faecal microbiome analysis using QIIME2 diversity plugins. The raw Illumina sequence data generated in this study are available in the Sequence Read Archive (SRA) at the National Center for Biotechnology Information (NCBI) under accession No. (SRP357402). All the data were released publicly.

Representative sequences for ASVs were built into a phylogenetic tree using phylogeny plugin (align-to-tree-mafft-fasttree pipeline) in QIIME2 and were taxonomically assigned using the Silva database (release 132) [49]. For determination of the α-diversity, the Shannon index values and observed ASVs were evaluated in QIIME2 using diversity plugin (core-metrics-phylogenetic pipeline) and visualized using GraphPad Prism 8. To test the variation in the gut microbiota, Jaccard distances between samples were evaluated and PCoA (principal coordinate analysis) based on Jaccard distances were performed in QIIME2 using diversity plugin (core-metrics-phylogenetic pipeline). The results were visualized with GraphPad Prism 8 to show the variations in the gut microbiota structure in mice. The differences in microbiome Shannon index, observed ASVs and principal coordinate variables among the groups were tested using Kruskal–Wallis test with Dunn’s multiple comparisons test with GraphPad Prism 8 software. LEfSe (http://huttenhower.sph.harvard.edu/galaxy/) was used to select ASVs with significant differences between two groups. The differential ASVs between maternal mice were visualized in heatmap using R package pheatmap (https://cran.r-project.org/web/packages/pheatmap/index.html.) The distribution of maternal core gut microbiota in all the groups was visualized also using R package pheatmap. Venn diagrams were generated using an online tool (http://bioinformatics.psb.ugent.be/webtools/Venn/).

### Metabolomic analysis

Analyses were performed using an ultrahigh performance liquid chromatography (UHPLC) system (1290 Infinity LC, Agilent Technologies) coupled to a quadrupole time-of-flight instrument (AB Sciex TripleTOF 6600). For hydrophilic interaction liquid chromatography (HILIC) separation, samples were analysed using a 2.1 mm ×100 mm ACQUITY UPLC BEH 1.7 μm column (Waters, Ireland). In both electrospray ionization (ESI) positive and negative ion modes, the mobile phases were as follows: A=25 mM ammonium acetate and 25 mM ammonium hydroxide in water, and B=acetonitrile. In auto tandem mass spectrometry (MS/MS) acquisition, the instrument was set to acquire over the m/z range of 25–1000 Da, and the accumulation time for the production scan was set at 0.05 s/spectrum. The production scan was acquired using information-dependent acquisition (IDA) with high-sensitivity mode selected. The collision energy (CE) was fixed at 35 V with ±15 eV. The declustering potential (DP) was set at ±60 V [50].

The raw MS data (wiff. scan files) were converted to MzXML files using ProteoWizard MSConvert and processed using XCMS for feature detection, retention time correction and alignment. The metabolites were identified by accuracy mass (<25 ppm) and MS/MS data, which were matched with our standards database. First, orthogonal partial least squares discrimination analysis (OPLS-DA) was performed on the metabolites detected in positive ion mode and negative ion mode respectively by SIMCA-P software (version 1.6.1). Then, metabolites detected in positive ion mode and negative ion mode were merged, and the following analysis were all based on the merged data. OPLS-DA of merged data was performed using R package ropls to test the variation of the faecal metabolites of mice between different groups, and the variable influence on projection (VIP) values of each metabolite in the model was obtained. Wilcoxon rank sum tests adjusted with original FDR method of Benjamini and Hochberg of the faecal metabolites between different groups were performed using R package MASS. The fold changes (log2 transformed) of metabolites between the groups were calculated in R 3.6.3. Metabolites that meet the conditions that VIP in OPLS-DA model > 1, |fold change (log2 transformed) |>1 and FDR < 0.05 were considered as the significant different metabolites between the groups.

### Clustering of co-abundant ASVs and metabolites

All the different ASVs identified between groups were clustered into co-abundance groups (CAGs) based on their SparCC correlations using the Ward clustering algorithm via the R package WGCNA [51]. The SparCC correlation coefficients of the ASVs were calculated using FastSpar (https://github.com/scwatts/FastSpar). Clusters were identified with the dynamic hybrid tree-cutting algorithm using a deepSplit of 2 and a minimum cluster size of 5 for clusters.

All the differential metabolites between groups were clustered into modules also using the R package WGCNA. Signed, weighted metabolite co-abundance correlation networks were calculated for all examined offspring mice in WGCNA using bicor() function [52]. A scale-free topology criterion was used to choose the soft threshold β = 12 for metabolites correlations. Clusters were identified with the dynamic hybrid tree-cutting algorithm using a deepSplit of 4 and a minimum cluster size of 5 for clusters. And the Hub ASV and metabolite was defined as the ASV or metabolite with highest module membership (MM) value in each CAG or module.

### Multi-omics correlation analysis

The spearman correlation coefficients of the differential ASVs, differential faecal metabolites and clinical parameters were calculated using MATLAB R2019b using corr() function. The *P-*values of the correlations were adjusted to FDR values with original FDR method of Benjamini and Hochberg. And the correlations were defined as significant when FDR < 0.05. The visual presentation of multiple omics correlations as performed using online tool Charticulator (https://charticulator.com/app/index.html).

## Supplementary Information

**Additional file 1: Figure S1.** The effect of a high-fat diet on the α-diversity of the gut microbiota in maternal mice during pregnancy and lactation. a Diversity, b Richness. The means are shown. * *P* < 0.05, tested by unpaired t test with Welch's correction. c Principal coordinate analysis (PCoA) of the maternal mice on the first two principal coordinates was performed based on the Jaccard distance. Comparisons were performed using the Kruskal–Wallis test for significant differences, *P<0.05, **P<0.01, ***P<0.001. Abbreviations: P, pregnancy; A, antepartum; L, lactation. The numbers indicate days. mHF (*n*=7): maternal group with high-fat diet; mNC (*n*=8): maternal group with normal control diet.

**Additional file 2: Figure S2.** The effect of high-fat diet consumption on the gut microbiota composition in maternal mice during pregnancy and lactation. The heatmap represents the normalized and log2-transformed relative abundances of the 128 ASVs that were significantly differentially abundant between maternal mice fed a NCD (mNC) and maternal mice fed a HFD (mHF). mHF (*n*=7): maternal group with high-fat diet; mNC (*n*=8): maternal group with normal control diet.

**Additional file 3: Figure S3.** Blocking microbial transmission altered the α-diversity of gut microbiota in offspring. a-c α-diversity, d-f, Richness. Comparisons were performed using the Kruskal–Wallis test for significant differences, and the P value was controlled at 0.05. a and d Offspring delivered by C-section versus offspring delivered via natural birth. b and e Offspring fed by their biological mothers versus offspring fed by cross-fostering. c and f Offspring delivered by C-section and fed by cross-fostering versus offspring born by natural birth and fed by their biological mothers. HF (*n*=10): offspring delivered by mHF group; NC (*n*=10): offspring delivered by mNC group. HF-CS (*n*=6): offspring born to mHF by C-section; NC-CS (*n*=10): offspring born to mNC by C-section. HF-CF (*n*=10): offspring delivered by mHF group and cross-fostered by mNC group; NC-CF (*n*=10): offspring delivered by mNC and cross-fostered by mHF group. HF-CS-CF (*n*=7); offspring born to mHF by C-section and cross-fostered by mNC group; NC-CS-CF (*n*=10): offspring born to mNC by C-section and cross-fostered by mHF group.

**Additional file 4: Figure S4.** Comparison of glycometabolic phenotypes among the NC-CS, NC-CF, and NC-CS-CF groups. a. Blood glucose levels by OGTT among the NC-CS, NC-CF, and NC-CS-CF groups. b Blood glucose levels by OGTT among the HF-CS, HF-CF, and HF-CS-CF groups. c Serum insulin levels during the OGTT among the NC-CS, NC-CF, and NC-CS-CF groups. d Serum insulin levels during the OGTT among the HF-CS, HF-CF, and HF-CS-CF groups. e Changes in blood glucose levels (% of initial) by the insulin tolerance test (ITT) among the NC-CS, NC-CF, and NC-CS-CF groups. f Changes in blood glucose levels (% of initial) by the insulin tolerance test (ITT) among the HF-CS, HF-CF, and HF-CS-CF groups. HF-CS (*n*=6): offspring born to mHF by C-section; NC-CS (*n*=10): offspring born to mNC by C-section. HF-CF (*n*=10): offspring delivered by mHF group and cross-fostered by mNC group; NC-CF (*n*=10): offspring delivered by mNC and cross-fostered by mHF group. HF-CS-CF (*n*=7); offspring born to mHF by C-section and cross-fostered by mNC group; NC-CS-CF (*n*=10): offspring born to mNC by C-section and cross-fostered by mHF group.

**Additional file 5: Figure S5.** Orthogonal projection to latent structure-discriminant analysis (OPLS-DA) score plots of faecal metabolites in offspring groups delineated by C-section intervention. a and c Comparisons under the negative ion mode. Each point represents one mouse.. b and d Comparisons under the positive ion mode. a and b Comparisons between offspring groups from healthy maternal mice. c and d Comparisons between offspring groups from maternal mice with hyperglycaemia during pregnancy. HF (*n*=10): offspring delivered by mHF group; NC (*n*=10): offspring delivered by mNC group. HF-CS (*n*=6): offspring born to mHF by C-section; NC-CS (*n*=10): offspring born to mNC by C-section.

**Additional file 6: Figure S6.** OPLS-DA score plots of faecal metabolites in offspring groups delineated by cross-fostering intervention. a and c Comparisons under the negative ion mode. b and d Comparisons under the positive ion mode. a and b Comparisons between offspring groups from healthy maternal mice. c and d Comparisons between offspring groups from maternal mice with hyperglycaemia during pregnancy. HF (*n*=10): offspring delivered by mHF group; NC (*n*=10): offspring delivered by mNC group. HF-CF (*n*=10): offspring delivered by mHF group and cross-fostered by mNC group; NC-CF (*n*=10): offspring delivered by mNC and cross-fostered by mHF group.

**Additional file 7: Figure S7.** OPLS-DA score plots of faecal metabolites in offspring groups delineated by C-section combined with cross-fostering intervention. a and c Comparisons under the negative ion mode. b and d Comparisons under the positive ion mode. a and b Comparisons between offspring groups from healthy maternal mice. c and d Comparisons between offspring groups from maternal mice with hyperglycaemia during pregnancy. HF (*n*=10): offspring delivered by mHF group; NC (*n*=10): offspring delivered by mNC group. HF-CS-CF (*n*=7); offspring born to mHF by C-section and cross-fostered by mNC group; NC-CS-CF (*n*=10): offspring born to mNC by C-section and cross-fostered by mHF group.

**Additional file 8: Table S1.**

## Data Availability

The data that support the findings of this study are available in the SRA at the NCBI with the identifiers SRP357402.

## Abbreviations

HIP: Hyperglycaemia in pregnancy
T2DM: Type 2 diabetes mellitus
HFD: High-fat diet
NCD: A normal chow diet
C-section, CS: Caesarean section
CF: Cross-fostering
SCFAs: Short-chain fatty acids
GDM: Gestational diabetes mellitus
PGDM: Pregestational diabetes
OGTT: Oral glucose tolerance test
AUC: Under the curve
ITT: Insulin tolerance test
GSIS: Glucose-stimulated insulin secretion test
PCoA: Principal coordinate analysis
ASVs: Amplicon sequence variants
OPLS-DA: Orthogonal partial least squares discriminant analysis
BCAAs: Branched-chain amino acids
FBG: Fasting blood glucose
2h-PG: 2 h postprandial glucose
LEfSe: Linear discriminant analysis effect size
SPF: Specific pathogen-free
BSA: Bovine serum albumin
GSI: Glucose stimulation index
ANOVA: One-way analysis of variance
PCR: Polymerase chain reaction
qPCR: Quantitative real-time PCR
SRA: Sequence Read Archive
UHPLC: Ultrahigh performance liquid chromatography
HILIC: Hydrophilic interaction liquid chromatography
ESI: Electrospray ionization
MS/MS: Mass spectrometry
IACUC: Institutional Animal Care and Use Committee
