# Supplementary figures and images for: Vertical transmission of the gut microbiota influences glucose metabolism in offspring of mice with hyperglycaemia in pregnancy
Source: Microbiome. 2022 Aug 9;10:122. doi: 10.1186/s40168-022-01318-8 (PMC9361546; doi:10.1186/s40168-022-01318-8)

**a**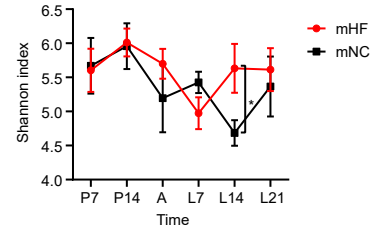**b**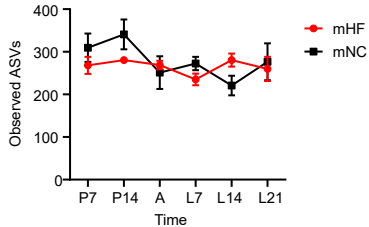**c**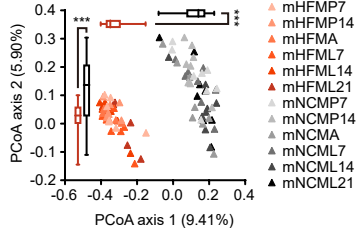

Supplement: Supplementary file 2 — Additional file 1: Figure S1. The effect of a high-fat diet on the α-diversity of the gut microbiota in maternal mice during pregnancy and lactation. a Diversity, b Richness. The means are shown. * P < 0.05, tested by unpaired t test with Welch's correction. c Principal coordinate analysis (PCoA) of the maternal mice on the first two principal coordinates was performed based on the Jaccard distance. Comparisons were performed using the Kruskal–Wallis test for significant differences, *P<0.05, **P<0.01, ***P<0.001. Abbreviations: P, pregnancy; A, antepartum; L, lactation. The numbers indicate days. mHF (n=7): maternal group with high-fat diet; mNC (n=8): maternal group with normal control diet. [file 40168_2022_1318_MOESM1_ESM.pdf]

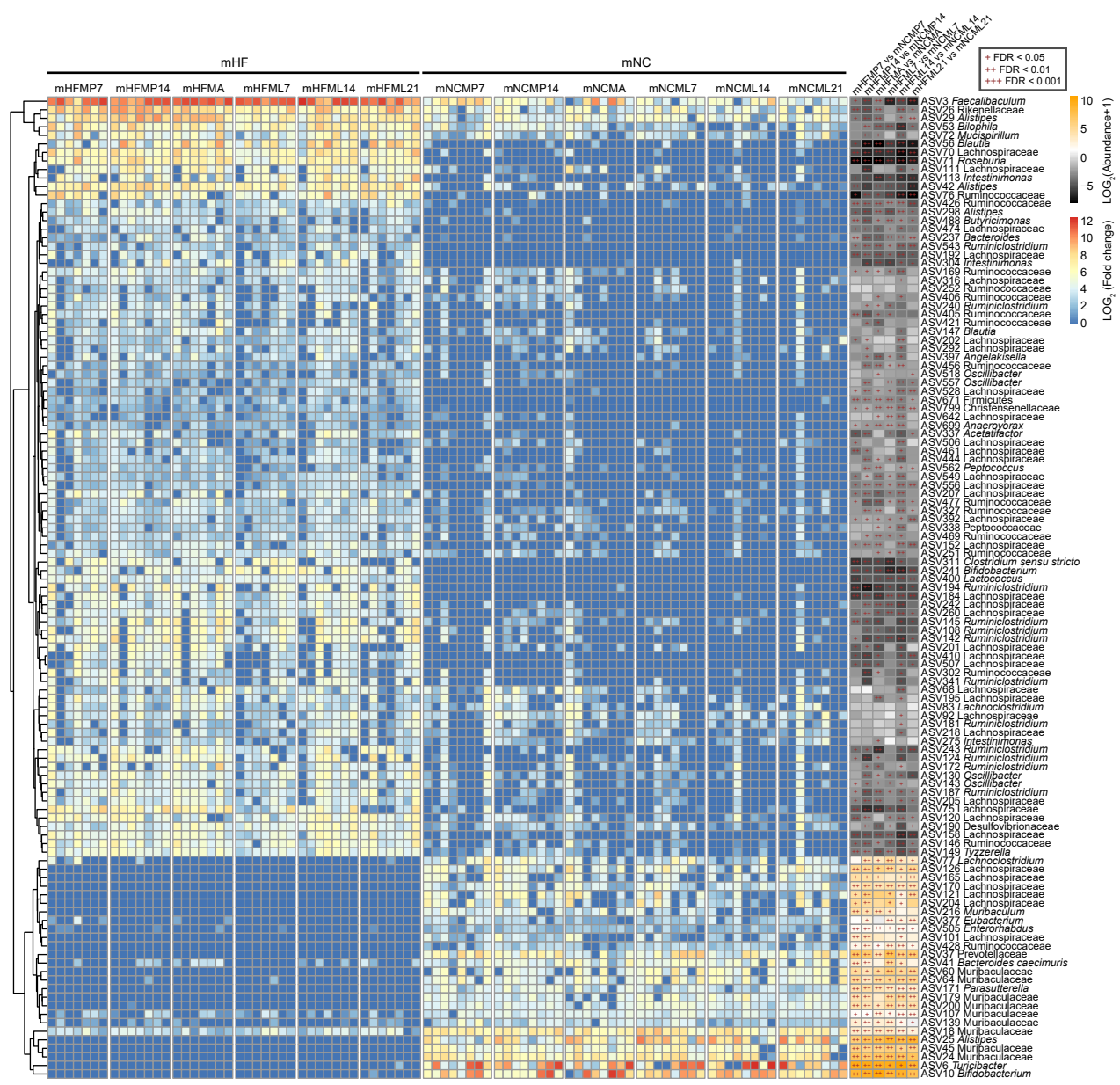

Supplement: Supplementary file 3 — Additional file 2: Figure S2. The effect of high-fat diet consumption on the gut microbiota composition in maternal mice during pregnancy and lactation. The heatmap represents the normalized and log2-transformed relative abundances of the 128 ASVs that were significantly differentially abundant between maternal mice fed a NCD (mNC) and maternal mice fed a HFD (mHF). mHF (n=7): maternal group with high-fat diet; mNC (n=8): maternal group with normal control diet. [file 40168_2022_1318_MOESM2_ESM.pdf]

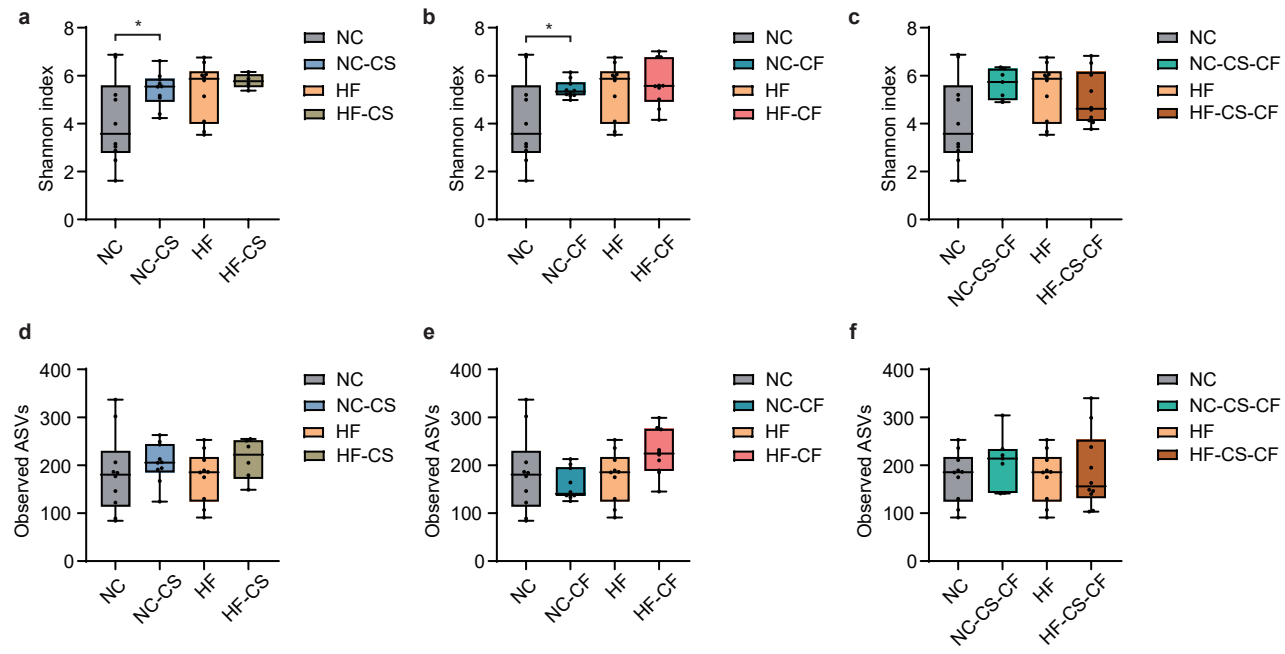

Supplement: Supplementary file 4 — Additional file 3: Figure S3. Blocking microbial transmission altered the α-diversity of gut microbiota in offspring. a-c α-diversity, d-f, Richness. Comparisons were performed using the Kruskal–Wallis test for significant differences, and the P value was controlled at 0.05. a and d Offspring delivered by C-section versus offspring delivered via natural birth. b and e Offspring fed by their biological mothers versus offspring fed by cross-fostering. c and f Offspring delivered by C-section and fed by cross-fostering versus offspring born by natural birth and fed by their biological mothers. HF (n=10): offspring delivered by mHF group; NC (n=10): offspring delivered by mNC group. HF-CS (n=6): offspring born to mHF by C-section; NC-CS (n=10): offspring born to mNC by C-section. HF-CF (n=10): offspring delivered by mHF group and cross-fostered by mNC group; NC-CF (n=10): offspring delivered by mNC and cross-fostered by mHF group. HF-CS-CF (n=7); offspring born to mHF by C-section and cross-fostered by mNC group; NC-CS-CF (n=10): offspring born to mNC by C-section and cross-fostered by mHF group. [file 40168_2022_1318_MOESM3_ESM.pdf]

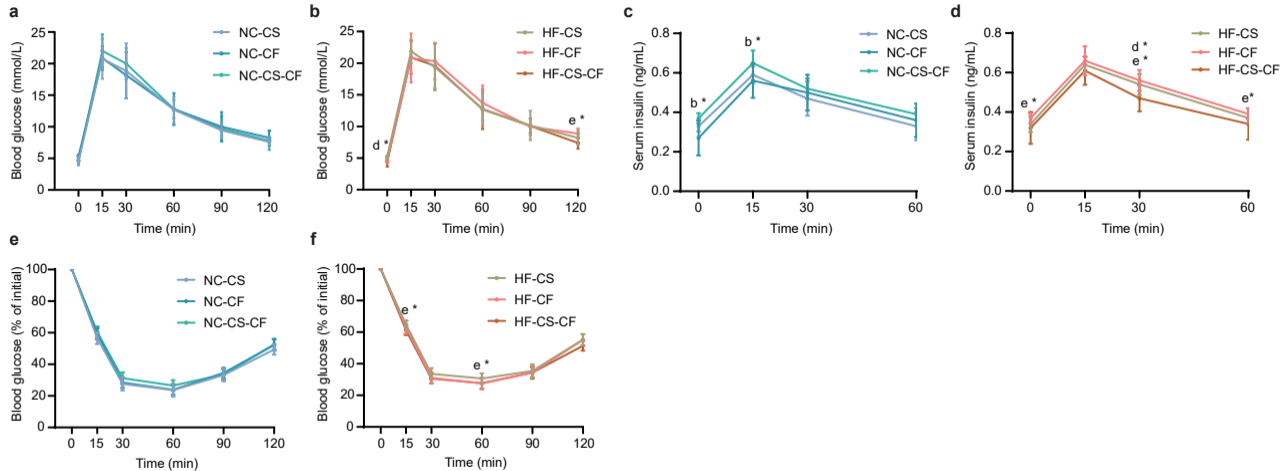

a: N-CS-CF vs N-CS ; b: N-CS-CF vs N-CF ; c: H-CS-CF vs H-CS ; d: H-CS-CF vs H-CF

Supplement: Supplementary file 5 — Additional file 4: Figure S4. Comparison of glycometabolic phenotypes among the NC-CS, NC-CF, and NC-CS-CF groups. a. Blood glucose levels by OGTT among the NC-CS, NC-CF, and NC-CS-CF groups. b Blood glucose levels by OGTT among the HF-CS, HF-CF, and HF-CS-CF groups. c Serum insulin levels during the OGTT among the NC-CS, NC-CF, and NC-CS-CF groups. d Serum insulin levels during the OGTT among the HF-CS, HF-CF, and HF-CS-CF groups. e Changes in blood glucose levels (% of initial) by the insulin tolerance test (ITT) among the NC-CS, NC-CF, and NC-CS-CF groups. f Changes in blood glucose levels (% of initial) by the insulin tolerance test (ITT) among the HF-CS, HF-CF, and HF-CS-CF groups. HF-CS (n=6): offspring born to mHF by C-section; NC-CS (n=10): offspring born to mNC by C-section. HF-CF (n=10): offspring delivered by mHF group and cross-fostered by mNC group; NC-CF (n=10): offspring delivered by mNC and cross-fostered by mHF group. HF-CS-CF (n=7); offspring born to mHF by C-section and cross-fostered by mNC group; NC-CS-CF (n=10): offspring born to mNC by C-section and cross-fostered by mHF group. [file 40168_2022_1318_MOESM4_ESM.pdf]

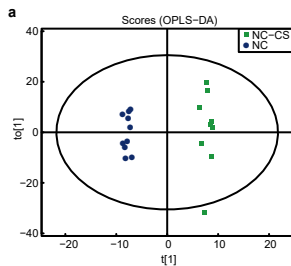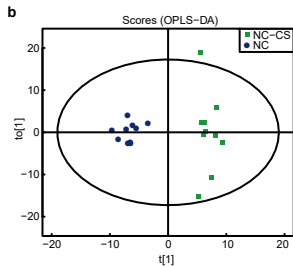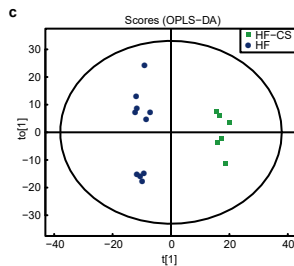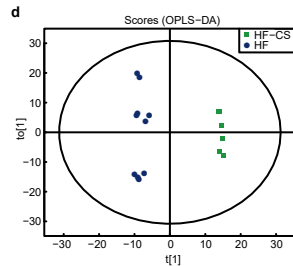

Supplement: Supplementary file 6 — Additional file 5: Figure S5. Orthogonal projection to latent structure-discriminant analysis (OPLS-DA) score plots of faecal metabolites in offspring groups delineated by C-section intervention. a and c Comparisons under the negative ion mode. Each point represents one mouse.. b and d Comparisons under the positive ion mode. a and b Comparisons between offspring groups from healthy maternal mice. c and d Comparisons between offspring groups from maternal mice with hyperglycaemia during pregnancy. HF (n=10): offspring delivered by mHF group; NC (n=10): offspring delivered by mNC group. HF-CS (n=6): offspring born to mHF by C-section; NC-CS (n=10): offspring born to mNC by C-section. [file 40168_2022_1318_MOESM5_ESM.pdf]

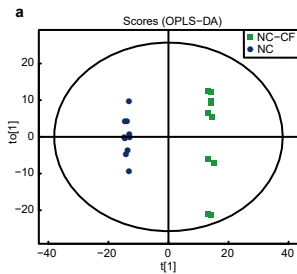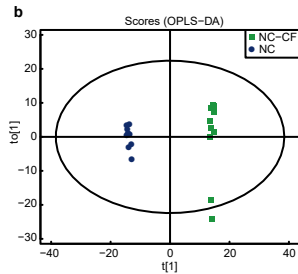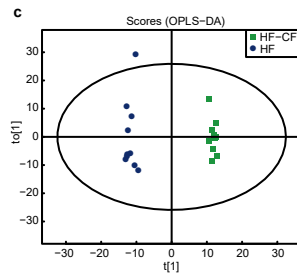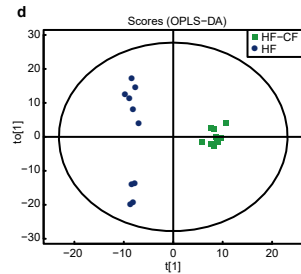

Supplement: Supplementary file 7 — Additional file 6: Figure S6. OPLS-DA score plots of faecal metabolites in offspring groups delineated by cross-fostering intervention. a and c Comparisons under the negative ion mode. b and d Comparisons under the positive ion mode. a and b Comparisons between offspring groups from healthy maternal mice. c and d Comparisons between offspring groups from maternal mice with hyperglycaemia during pregnancy. HF (n=10): offspring delivered by mHF group; NC (n=10): offspring delivered by mNC group. HF-CF (n=10): offspring delivered by mHF group and cross-fostered by mNC group; NC-CF (n=10): offspring delivered by mNC and cross-fostered by mHF group. [file 40168_2022_1318_MOESM6_ESM.pdf]

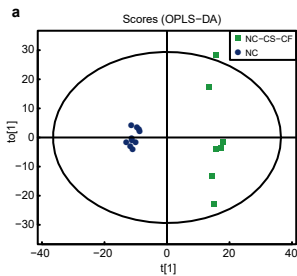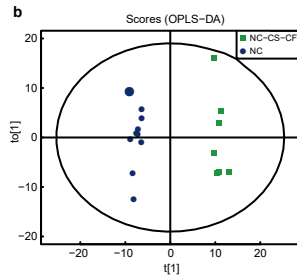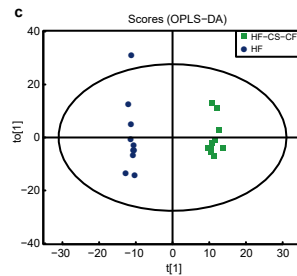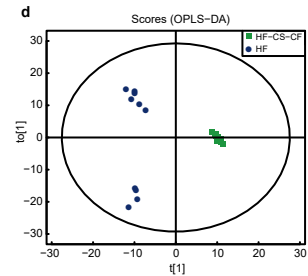

Supplement: Supplementary file 8 — Additional file 7: Figure S7. OPLS-DA score plots of faecal metabolites in offspring groups delineated by C-section combined with cross-fostering intervention. a and c Comparisons under the negative ion mode. b and d Comparisons under the positive ion mode. a and b Comparisons between offspring groups from healthy maternal mice. c and d Comparisons between offspring groups from maternal mice with hyperglycaemia during pregnancy. HF (n=10): offspring delivered by mHF group; NC (n=10): offspring delivered by mNC group. HF-CS-CF (n=7); offspring born to mHF by C-section and cross-fostered by mNC group; NC-CS-CF (n=10): offspring born to mNC by C-section and cross-fostered by mHF group. [file 40168_2022_1318_MOESM7_ESM.pdf]
